# Emicizumab is well tolerated and effective in people with congenital hemophilia A regardless of age, severity of disease, or inhibitor status: a scoping review

**DOI:** 10.1016/j.rpth.2024.102415

**Published:** 2024-04-18

**Authors:** Guy Young, Steven W. Pipe, Gili Kenet, Johannes Oldenburg, Mariam Safavi, Tuende Czirok, Francis Nissen, Johnny Mahlangu

**Affiliations:** 1 Cancer and Blood Disorders Institute, Children's Hospital Los Angeles, University of Southern California Keck School of Medicine, Los Angeles, California, USA; 2 Departments of Pediatrics and Pathology, University of Michigan, Ann Arbor, Michigan, USA; 3 The Israeli National Hemophilia Center and Thrombosis Unit, Sheba Medical Center, Tel Hashomer, Israel; 4 The Amalia Biron Thrombosis Research Institute, Tel Aviv University, Tel Aviv, Israel; 5 Institute of Experimental Hematology and Transfusion Medicine, University Clinic Bonn, Bonn, Germany; 6 Product Development, F. Hoffmann-La Roche Ltd, Basel, Switzerland; 7 Department of Molecular Medicine and Haematology, School of Pathology, Faculty of Health Sciences, University of the Witwatersrand and National Health Laboratory Service, Johannesburg, Gauteng, South Africa

**Keywords:** clinical trials, emicizumab, hemophilia A, quality of life, real-world experience, review, safety

## Abstract

**Background:**

With the treatment landscape continually evolving, it is vital that the hemophilia community have an overview of all published data for approved therapies, such as emicizumab, to support shared decision making.

**Objectives:**

To bring together the clinical and real-world data for emicizumab use in people with congenital hemophilia A, regardless of age, disease severity, or factor VIII inhibitor status. Key focus areas were safety, efficacy, and quality of life (QoL).

**Methods:**

This scoping review used citation databases (PubMed, Embase, and the Cochrane Library) and manual searches of abstract books. Publications reporting original data for emicizumab in people with hemophilia A, published in English after December 2014, and reporting select endpoints were included. This narrative synthesis focused on zero bleeds, treated annualized bleeding rate (ABR), adverse events, and QoL measures.

**Results:**

Overall, 97 publications were included (cut-off: August 9, 2022). Treated ABR remained low (calculated mean and median treated ABRs ranged between 0.7-1.3 and 0.0-1.4, respectively), and the median percentage of people with zero treated bleeds was 66.7%. The proportion of people experiencing treatment-related adverse events ranged from 0.0% to 60.0%; most were injection-site reactions. Across 37 publications reporting on safety and enrolling >2300 individuals, 11 thrombotic events and 4 thrombotic microangiopathies were reported. Data from well-established tools show QoL benefits with emicizumab.

**Conclusion:**

This scoping review consolidates the global published experience for emicizumab in people with hemophilia A and supports the fact that emicizumab has an acceptable safety profile, is effective and efficacious in bleed prevention, and is associated with improvements in QoL.

## Essentials

• Emicizumab was first approved for hemophilia A in 2017, and over 20,000 people have been treated.
• An overview of emicizumab experience may help physicians and patients with treatment decisions.
• This scoping review summarizes the clinical and real-world data for emicizumab in one article.
• Data support the safety profile, bleed prevention, and improved quality of life with emicizumab.

## Graphical abstract

**Figure:**
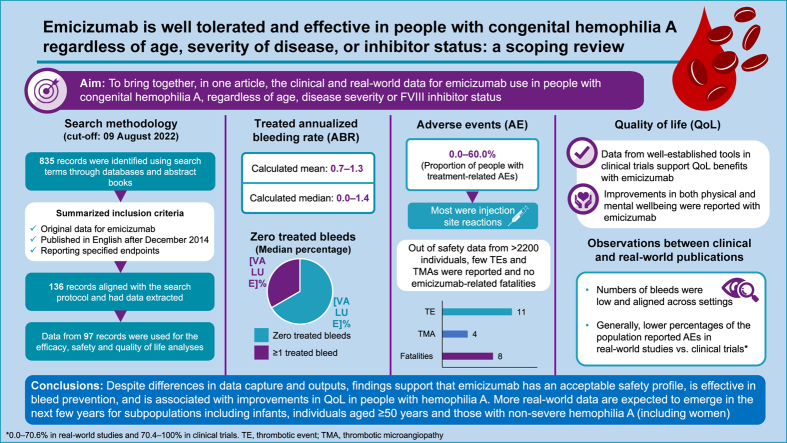

## 1 Introduction

With the treatment landscape for congenital hemophilia A continually evolving [1], the medical community and those with hemophilia A must be provided with a comprehensive overview of the efficacy and safety data for approved therapy options to support informed decision making. Prophylaxis with replacement clotting factors has long been the standard of care for people with severe hemophilia A to prevent bleeding [2]. Over the last decade, extended half-life factor (F)VIII therapies, for example, efanesoctocog alfa (mean half-life of 47 hours), have been developed to reduce treatment burden, but these still require frequent intravenous infusions [3,4], and up to 30% of those receiving FVIII replacement therapy develop FVIII inhibitors [5,6].

Recently, the innovative FVIII mimetic, emicizumab, was developed with the aim of addressing the unmet needs in hemophilia A. Emicizumab was approved for use in the United States in 2017 [7], Europe and Japan in 2018 [8,9], and subsequently by many other countries. Emicizumab is a bispecific antibody that is administered subcutaneously; it works by mimicking the cofactor function of FVIII by bridging activated FIX and FX [10]. It shares no sequence homology with FVIII, meaning it can restore hemostasis regardless of the presence of FVIII inhibitors [11]. Additionally, emicizumab has a half-life of 26.8 days, which permits sustained activity levels with less frequent and flexible dosing [8,11], resulting in an improved adherence to therapy compared with factor replacement products [12]. The safety and efficacy of emicizumab have been demonstrated in people with congenital hemophilia A from infancy to adulthood, with and without inhibitors, in the HAVEN clinical trial program [[13], [14], [15], [16]] and many real-world studies [[17], [18], [19], [20]].

The global experience with emicizumab is accumulating rapidly, with over 20,000 people having been treated as of March 2023 [21], including specific patient populations (infants [22], individuals aged ≥50 years [23], and those with non-severe hemophilia A [24]). A single source of all data from the clinical and real-world settings for emicizumab is currently lacking; such a resource may help educate healthcare providers and patients and support informed shared decision making.

## 2 Methods

### 2.1 Search methodology

This scoping review utilized the 5-stage methodological framework described by Arksey and O’Malley [25]. The Preferred Reporting Items for Systematic Reviews and Meta-Analyses extension for scoping reviews was also followed (Supplementary Table S1). The search query was designed in conjunction with an information professional; it consisted of index and free-text terms (Supplementary Table S2), translated into the syntax for each database.

A comprehensive search of the literature was conducted on August 9, 2022, using the citation databases PubMed (manuscript records), Embase (manuscript records and congress abstracts), and the Cochrane Library (for manuscripts, abstracts, and clinical trial records). Additionally, manual searches of abstract books for relevant congresses were performed (Supplementary Table S3).

### 2.2 Eligibility criteria for included publications

Inclusion criteria were developed using the Population, Intervention, Comparison, and Outcome model (Table 1); information on exclusion criteria is available in the footnote. No limits were placed on the study design.

**Table 1:** Population, Intervention, Comparison, and Outcome criteria.

PICO	Inclusion criteria
Population	Individuals with congenital hemophilia A, of all ages and severities, with and without inhibitors against FVIII
Intervention	Prophylaxis with emicizumab
Comparison	Studies with and without comparison groups were included
Outcomes	At least 1 of the following: bleeds (ABR, zero bleeds, joint bleeds, treated bleeds, spontaneous bleeds, and any other bleeds reported), including their location and severity; safety (AEs, serious AEs, treatment-related AEs, thrombotic events, and thrombotic microangiopathy); immunogenicity, including the suspected and/or confirmed presence of ADAs and any associated impact on emicizumab efficacy; target joints and overall joint health (measured using the HJHS or another tool); physical activity; QoL (measured using a QoL questionnaire and/or the SQ-ISHI); social impact (such as no. of days missed at school/work and mental health); surgical outcomes; and other relevant parameters, such as pain, adherence, and treatment burden (no. of injections)

Additional inclusion criteria: only reports published after 2014 were included, as all studies involving emicizumab were conducted after this date. Primary research in the English language from any income country and with extractable data was included. Scientific Congress abstracts were included if they met the inclusion criteria.

Exclusion criteria: narrative reviews, editorials, biographies, comments, and previous systematic reviews. Studies focusing on inherited or acquired bleeding disorders other than congenital hemophilia A and those reporting on *in vitro* models, animal models, cell lines, and other molecules were excluded.

ABR, annualized bleeding rate; ADA, antidrug antibody; AE, adverse event; HJHS, Hemophilia Joint Health Score; PICO, Population, Intervention, Comparison, Outcomes; QoL, quality of life; SQ-ISHI, Satisfaction Questionnaire with Intravenous or Subcutaneous Haemophilia Injection.

Studies with <5 patients and case reports on individual patients were generally excluded to avoid skewing the overall data. However, if case series/reports described rare adverse events (AEs) constituting valuable information, eg, cases of antidrug antibodies, severe bleeding, or thrombotic events following emicizumab prophylaxis, they were reported separately in the results (see section 3.4; not part of the overall efficacy and safety data). Similarly, case studies with <5 patients were only considered in populations where data are scarce (previously untreated patients [PUPs]/minimally treated patients [MTPs]; infants; people aged ≥50 years; people with nonsevere disease); these findings are reported separately as part of a subanalysis (section 3.6).

### 2.3 Screening and data extraction

The database screening and data extraction processes were performed using the Covidence systematic review platform (Veritas Health Innovation). All texts underwent independent screening by 2 reviewers according to the eligibility criteria. A third reviewer resolved conflicts. Data were extracted by 2 separate people in Covidence, and a third person performed a consensus check.

### 2.4 Outcomes

Captured outcomes are reported in Table 2. The efficacy, effectiveness, and safety outcomes were explored in different populations of interest, as identified in section 2.2. Data from quality of life (QoL) tools were also captured in a narrative format. Additional outcomes captured in the search, such as joint bleeds, spontaneous bleeds, target joints, overall joint health, physical activity, surgical outcomes, and adherence, are not reported here.

**Table 2:** Captured outcomes in this scoping review.

Captured outcomes
Efficacy outcomes
Proportion of people with zero bleeds
Treated bleeds
All bleeds
ABR for treated bleeds
Model-based^a^
Calculated mean
Calculated median
Safety outcomes
No. and percentage of participants with:
≥1 AE
Serious AEs
Treatment-related AEs
Local ISR
Thrombotic events
Thrombotic microangiopathy events
Fatalities
Immunogenicity outcomes
No. and percentage of individuals with:
Suspected or confirmed ADAs
ADAs associated with loss of emicizumab efficacy

ABR, annualized bleeding rate; ADA, antidrug antibody; AE, adverse event; ISR, injection-site reaction.

a The model-based ABR was a binomial regression model, which took into account variations in the efficacy follow-up period, most commonly used in clinical trials versus real-world studies.

### 2.5 Analysis

In the case of multiple publications reporting outcomes for the same population, studies with the most recent data cut-off were included to avoid duplication. The only exception was for efficacy outcomes in the HAVEN 1 to 4 clinical trials; the primary publications were used as the reporting methods were more consistent with other studies, ie, the most recent publication by Callaghan et al. [13] reported results over 24-week intervals rather than 1 single time point (typical of all other publications). Data from each study/publication were captured as reported: in clinical trials, results were presented per arm; however, in the case of real-world studies, the overall results for the population were reported. Owing to the broadness and variety of the literature search results, statistical analysis was limited to reporting a range of values and calculating the mean, median, and SD. For annualized bleeding rate (ABR), only the range and median values of the collective model-based ABR, calculated mean, and median ABR were reported. Different types of ABR and zero bleed outcomes are usually reported separately in clinical trials (ie, all bleeds, treated bleeds, spontaneous bleeds, and joint bleeds) versus real-world studies, which typically report on just “ABR” or “zero bleeds” with no specification if the bleeds were treated, spontaneous, or occurred specifically in the joint. As such, “ABR” or “zero bleeds” in real-world publications were classed as “all bleeds” in this scoping review unless they specified the type of bleed. Since the observation time for zero bleed data varied considerably across studies, and follow-up time is of particular importance for the zero bleed outcomes analysis, a very broad range of time in weeks/months for clinical trials and real-world data was noted to contextualize the results. A sensitivity analysis was conducted to take into consideration the heterogeneity introduced by studies with a small number of participants (<10) and the different types of zero bleeds reported. The analysis was restricted to studies with a larger sample size (≥10 participants) and limited the different types of zero bleeds reported in real-world studies to “zero treated bleeds” and “zero all bleeds,” ie, studies only reporting on zero spontaneous bleeds were not included. Owing to the wide range of publications in this scoping review, a narrative synthesis, constituting descriptive text, was used to explain the findings.

## 3 Results

### 3.1 Descriptive characteristics

**Figure 1:**
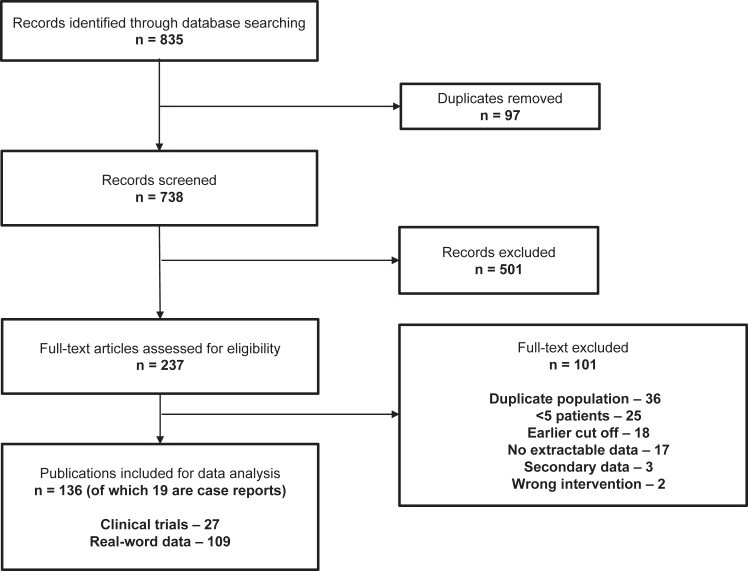
Preferred Reporting Items for Systematic Reviews and Meta-Analyses (PRISMA) flow diagram of identified references.

Overall, 835 publications were identified through the database and manual searches (Figure 1). Following screening, 136 publications aligned with the search protocol: 27 on clinical trials, 109 on real-world studies, and 19 case reports. Out of 136 publications, 97 were used for the efficacy, safety, and QoL analyses; the remaining 39 focused on joint health, physical activity, surgical outcomes, and adherence and will be analyzed and reported separately. The number of publications for different populations is shown in Table 3. The number of patients analyzed for each outcome is included throughout the text.

**Table 3:** Search outputs for different hemophilia populations.

Population	No. of publications
Inhibitors against FVIII	
People with inhibitors against FVIII	37
People without inhibitors against FVIII	24
People with or without inhibitors against FVIII	62
Age groups	
PUP	11
Infants (≤12 mo)	24
Pediatric (1 to <18 y)	101
Adults (≥18 y)	91
Adults (≥50 y of age)	31
Severity of hemophilia (FVIII activity)	
Mild (>5% to <40%)	18
Moderate (≥1% to ≤5%)	26
Severe (<1%)	76

FVIII, factor VIII; PUP, previously untreated patient.

### 3.2 Efficacy

#### 3.2.1 ABRs for treated bleeds

The focus here will be on calculated ABR (mean and median) as this is reported in both study settings; model-based ABR values and ranges reported in clinical trials are summarized in Table 4.

**Table 4:** Summary of treated bleed data in the total population.

	Clinical trials	Real-world data studies	All studies
Calculated mean ABR for treated bleeds			
Range	0.7-1.3	0.2-1.4	0.2-1.4
Median	0.9	0.4	0.6
No. of publications	2	5	7
Sample size	85	570	655
Calculated median ABR for treated bleeds			
Range	0.0-1.4	0.0-1.0	0.0-1.4
Median	0.0	0.1	0.0
No. of publications	7	6	13
Sample size	604	503	1107
Model-based ABR for treated bleeds^a^			
Range	0.2-5.1	-	-
Median	1.3	-	-
No. of publications	9	-	-
Sample size	680	-	-
Zero treated bleeds			
Range, %	33.3-82.6^b^	50.8-85.7	33.3-85.7
Adjusted range, % (sensitivity analysis)	55.6-82.6	50.8-85.7	55.6-85.7
Median, %	62.9	70.5	66.7
Adjusted median, % (sensitivity analysis)	62.9	70.5	65.5
No. of publications	9	6	15
Sample size	680	213	893

ABR, annualized bleeding rate; Q2W, every 2 weeks; Q4W, every 4 weeks.

a Determined using negative binomial regression to account for different follow-up times, but only reported in clinical trials as the format of data collected in real-world studies does not usually allow for estimates to be made using this method.

b It must be noted that the 33.3%, at the lower end of the range for zero treated bleeds in clinical trials, comes from a small subgroup of participants in HOHOEMI [16,26] who received emicizumab every 2 weeks; 2 of the 6 participants had zero treated bleeds and out of 6 bleeds that occurred in 4 patients, only 1 treated bleed was spontaneous, and the other 5 treated bleeds were traumatic. It is noted by Shima et al. [16,26] that physical activity in these pediatric patients shifted toward moderate-to-high-risk activity during treatment, which may explain the number of traumatic bleeds observed. These data were excluded from the sensitivity analysis, bringing the range up to 55.5% to 82.6% for clinical trials, and when both Q2W and Q4W cohort data are pooled (*n* = 13), results for zero treated bleeds are consistent with other HAVEN studies (*n* = 7/13; 53.8%).

The mean calculated ABR for treated bleeds ranged from 0.7 to 1.3 (*n* = 85) in clinical trials and from 0.2 to 1.4 in real-world studies (*n* = 570); the median values were 0.9 and 0.4, respectively (Table 4). The range for median calculated ABR for treated bleeds was 0.0 to 1.4 in clinical trials (*n* = 604) and 0.0 to 1.0 in real-world studies (*n* = 503), with the median values being 0.0 and 0.1, respectively (Table 4 and Supplementary Tables S4 and S5).

#### 3.2.2 Zero treated bleeds

In clinical trials, the follow-up time for assessment of zero treated bleeds ranged from ∼19 weeks to ∼2 years, and the observation times in real-world studies were similarly variable, ranging from ∼6 months to ∼2.4 years. Whilst no statistical analysis was performed, no trend toward a lower percentage of people with zero bleeding events with increasing follow-up time was observed.

**Figure 2:**
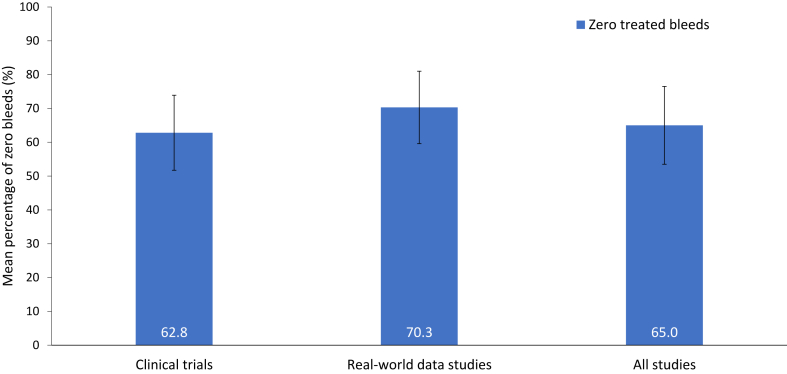
Percentage of zero treated bleeds in people with congenital hemophilia A receiving emicizumab prophylaxis in clinical trials and real-world studies. Error bars indicate the SD of the mean. Clinical trial data are based on 680 participants from 9 publications for zero treated bleeds (range, 33.3%-82.6%). Real-world data are based on 213 participants from 6 publications for zero treated bleeds (range, 50.8%-85.7%).

Overall, mean and median percentages of zero treated bleed outcomes remained above 62% and were slightly higher in the real-world setting (Table 4 and Figure 2). The mean percentage of people with zero treated bleeds in clinical trials was 62.8% (range, 33.3%-82.6%; *n* = 680; see Table 4 for more information on the range) and 64.4% (range, 55.6%-82.6%; *n* = 667) when adjusted in the sensitivity analysis. In real-world studies, the mean percentage of people with zero treated bleeds was 70.3% (range, 50.8%-85.7%; *n* = 230) and 69.4% (range, 50.8%-85.7%; *n* = 198) following the sensitivity analysis. The median percentage of people with zero treated bleeds in clinical trials and real-world studies was 62.9% and 70.5%, respectively. Data on zero all bleeds can be found in Table 4.

### 3.3 Safety outcomes

The percentage of clinical trial participants experiencing ≥1 AE ranged from 70.4% to 100% (*n* = 677); a wider range (0.0%-70.6%) was observed for real-world studies (*n* = 1055; Table 5 and Supplementary Tables S6 and S7). The percentage of people with hemophilia A experiencing serious AEs in the clinical trial (*n* = 677) and real-world (*n* = 291) settings ranged from 0.0% to 30.0%; relatedness to emicizumab was not consistently reported. In clinical trial participants (*n* = 677), the reported percentage range of people with treatment-related AEs was 0% to 60.0% (median, 29.0%). The most common treatment-related AE was injection-site reaction (ISR). A lower range of treatment-related AEs (0.0%-13.0%) was reported in real-world studies (median, 0.0%; *n* = 455), and the percentage of the population (*n* = 930) reporting local ISRs was 0% to 10.7%. In 41 publications (*n* = 2363) reporting on thrombotic events, the total number of events was 11 (5 in clinical trials and 6 in real-world data studies), and the percentage of the population experiencing a thrombotic event ranged from 0% to 3.6% (*n* = 2363). In HAVEN 1, 2 thrombotic events occurred in participants who received a cumulative dose of >100 U/kg/24 h of activated prothrombin complex concentrate (APCC) for ≥24 hours [10]. Three additional thrombotic events were reported in clinical trials (1 in HAVEN 6 [27] and 2 in STASEY [28]) in people with known comorbidities or pre-existing risk factors. In real-world studies, 6 thrombotic events were reported: 1 case was associated with emicizumab and APCC use [29]; 1 patient developed a cephalic vein thrombophlebitis after a surgical procedure (for which he received factor replacement) [30]; 1 patient with severe symptomatic ischemic heart disease and a port infection had a non-ST elevation myocardial infarction [31]; 1 patient with atherosclerosis with aortic calcification had a chronic superior mesenteric artery thrombosis [31]; 1 patient had an atypical cardiac event that led to cessation of emicizumab [31]; and 1 infant presented with central venous line thrombosis [32]. Three cases of thrombotic microangiopathy events were reported in clinical trials, and one other case was reported in the real-world setting; these were in association with APCC use of >100 U/kg/24 h for ≥24 hours (emicizumab was discontinued 1 month preoperatively in the real-world case) [33].

**Table 5:** Summary of safety outcomes in the total population.

	Clinical trials	Real-world data studies	All studies
Percentage of population with ≥1 AE			
Range	70.4-100	0.0-70.6	0.0-100
Mean (SD)	87.2 (9.6)	10.2 (18.1)	50.0 (41.0)
Median	86.2	2.0	70.6
No. of publications	9	14	23
Sample size	677	1055	1732
Percentage of population with ≥1 serious AE			
Range	2.0-30.0	0.0-20	0.0-30.0
Mean (SD)	11.9 (7.2)	2.3 (6.3)	8.3 (8.3)
Median	11.1	0.0	7.6
No. of publications	9	9	18
Sample size	677	291	968
Percentage of population with treatment-related AEs			
Range	0-60.0	0.0-13.0	0.0-60.0
Mean (SD)	28.4 (13.5)	2.4 (4.8)	21.0 (16.6)
Median	29.0	0.0	20.0
No. of publications	9	6	15
Sample size	677	455	1132
Percentage of population with local ISR			
Range	0.0-60.0	0.0-10.7	0.0-60.0
Mean (SD)	21.1 (12.9)	3.9 (4.2)	13.4 (13.2)
Median	20.0	1.6	10.2
No. of publications	9	12	21
Sample size	677	930	1607
Percentage of population with thrombotic events			
Range	0.0-2.9	0.0-3.6	0.0-3.6
Total no. of thrombotic events reported	5	6	11
No. of publications	9	32	41
Sample size	677	1686	2363
Percentage of population with thrombotic microangiopathy events			
Range	0.0-2.9	0.0-2.4	0.0-2.9
Total no. of thrombotic microangiopathy events reported	3	1	4
No. of publications	9	32	41
Sample size	677	1686	2363
Percentage of the population with a fatality			
Range	0.0-2.8	0.0-1.2	0.0-1.2
Total no. of fatalities reported	3	5	8
No. of publications	10	31	41
Sample size	696	1550	2246
Percentage of population with suspected/confirmed ADAs			
Range	5.1-22.2	0-9.1	0-22.2
No. of publications	2	9	11
Sample size	686	1255	1941
Percentage of population with ADAs with loss of efficacy			
Range	0-0.1	0-4.5	0-4.5
No. of publications	2	6	8
Sample size	686	334	1020

ADA, antidrug antibody; AE, adverse event; ISR, injection-site reaction.

Eight fatalities were reported across 41 studies (*n* = 2246) in clinical trials and real-world studies; none were reported to be related to emicizumab. Two were in the phase 3 STASEY trial (1 polytrauma with fatal head injury and 1 abdominal compartment syndrome) [15]. One death was reported in the HAVEN 1 study (rectal hemorrhage) [10]. Five fatalities were from real-world studies and registries, including hemorrhagic shock secondary to a presumed gastrointestinal bleed [34], a multiorgan failure complicating viral infection [31], intra-abdominal bleeding (with delayed presentation to the hospital) [31], retroperitoneal hematoma in a 5-month-old following concomitant anticoagulant therapy with low-molecular-weight heparin to treat a central venous line-related thrombosis [32], and 1 reported as unrelated problems [35].

Data on immunogenicity were available for 1941 people with hemophilia A across 11 publications; the range of people with suspected (*n* = 1) or confirmed antidrug antibodies (ADAs; *n* = 43) was 0% to 22.2%. Across 8 publications (*n* = 1020), the presence of ADAs with loss of efficacy ranged from 0% to 4.5%. Overall, in clinical trials, 1 out of 686 (0.14%) people with hemophilia A discontinued emicizumab due to loss of efficacy [26,36]. In 6 publications in the real-world setting, with a combined total of 334 people with hemophilia A, 2 individuals stopped emicizumab due to loss of efficacy [17,37].

### 3.4 Additional case reports and case series

#### 3.4.1 Safety

A total of 19 case studies reported safety events associated with emicizumab that were not already reported (Supplementary Table S8). Most events, excluding ADA development, were considered unrelated to emicizumab and successfully resolved. Unresolved cases included 1 report of post-immune tolerance induction inhibitor recurrence in a 10-year-old patient [38] and a second case where emicizumab treatment was stopped due to hematuria and a severe ISR reported to be associated with emicizumab [39].

Of note, other safety events reported as potentially being related to emicizumab included an ST-segment elevation myocardial infarction and pulmonary embolism [40], full-house lupus nephritis [41], and rhabdomyolysis [42]; all occurred in people with hemophilia A with inhibitors. All events were managed successfully, and no deaths were reported in case reports.

#### 3.4.2 Development of ADAs

Four real-world cases (all receiving emicizumab 1.5 mg/kg once weekly) described the development of neutralizing ADAs; emicizumab was discontinued in 2 of these reports [[43], [44], [45], [46]] (Supplementary Table S9). One additional case described ADAs in a 2-year-old boy with severe hemophilia A that left emicizumab activity unaffected but increased emicizumab clearance; hence, emicizumab was stopped after 3 months [47].

### 3.5 QoL

The tools used to measure QoL in clinical trials and real-world studies are shown in Table 6. The Hemophilia QoL Questionnaire for Adults (Haem-A-QoL) has demonstrated consistent benefits of emicizumab for people with hemophilia A [10,14,[48], [49], [50], [51], [52]]. In the HAVEN trials, benefits were particularly apparent in terms of Physical Health domain scores (maintained for >70 weeks) and the Treatment domain assessing treatment burden [48]. EQ-5D-5L Visual Analog and Index Utility scores also improved with emicizumab in the HAVEN study program. The only real-world Haem-A-QoL data for people with hemophilia A receiving emicizumab were obtained in a small study (*n* = 8 patients at 2 hospitals in Malaysia) and demonstrated significant (*P* ≤ .001) improvements across all domains vs prior to receiving emicizumab [49].

**Table 6:** Quality of life tools used in clinical trials and real-world publications.

Population	No. of publications
Clinical trial publications	
Haem-A-QoL	6
EQ-5D-5L	5
Haem-QoL SF	3
EmiPref	2
Adapted Inhib-QoL	2
SQ-ISHI	1
Other	2
Real-world publications	
pedHAL	2
Haem-A-QoL	1
Haem-QoL SF	1
EQ-5D-5L	1
HEMOCAB	1
PROBE	1
CATCH	1
Other	5

Adapted Inhib-QoL, Inhibitor-Specific Quality of Life with Aspects of Caregiver Burden; CATCH, Comprehensive Assessment Tool of Challenges in Hemophilia; EmiPref, Emicizumab Preference; EQ-5D-5L, EuroQoL-5 dimensions-5 levels; Haem-A-QoL, Haemophilia Quality of Life Questionnaire for Adults; Haem-QoL, Haemophilia Quality of Life Questionnaire; Haem-QoL SF, Haemophilia Quality of Life Questionnaire Short Form; HEMOCAB, HEMOphilia associated Caregiver Burden scale; pedHAL, Paediatric Haemophilia Activities List; PROBE, Patient Reported Outcomes, Burdens and Experiences; SQ-ISHI, Satisfaction Questionnaire with Intravenous or Subcutaneous Haemophilia Injection.

The Emicizumab Preference (EmiPref) survey highlighted high levels of patient preference for emicizumab compared with previous therapy, with 94% of 95 respondents reporting a preference for emicizumab in HAVEN 3 and 100% of 41 respondents reporting a preference for emicizumab in HAVEN 4 [53]. The Satisfaction Questionnaire with Intravenous or Subcutaneous Haemophilia Injection tool (SQ-ISHI), which incorporates the “Patient Global Impression of Change” item assessing treatment satisfaction, showed improvements in measures of confidence (+2.9 for “satisfaction with treatment half-life” and +2.0 for “overall satisfaction”) and reductions in measures of worry (−2.5 for “bother” and −1.8 for “travel impact”) for patients who switched to emicizumab in HAVEN 3 [54]. Similarly, in a self-designed questionnaire from Panovska-Stavridis et al. [55], 100% of patients (*n* = 10) were “satisfied” or “very satisfied” with emicizumab prophylaxis.

Joint pain has been measured using the Comprehensive Assessment Tool of Challenges in Hemophilia (CATCH) (*n* = 9) [56], where participants reported reduced pain with emicizumab, and a patient perception questionnaire regarding bleeding symptoms, joint symptoms, daily life, and feelings (*n* = 17) [26], where all but 1 participant reported “improved,” “slightly improved,” or “unchanged” pain after starting emicizumab.

### 3.6 Subgroup analyses

#### 3.6.1 Infants (≤12 months)

At the time of this literature search, data on infants in clinical trials with emicizumab prophylaxis were scarce. All publications focusing on the subgroups of interest are summarized in Table 7. While 2 clinical trials (HAVEN 2: *n* = 8; <2 years of age [57] and HOHOEMI: *n* = 1; 4 months of age at baseline [16]) included infants in the wider population, no data for infants (up to 12 months of age) were explicitly reported, except that the 4-month-old had no treated bleeds over 24 weeks of followup. Five real-world studies presented bleed data on 24 infants. In Hassan et al. [58] and Wieland et al. [59], all 9 infants had zero treated bleeds. In Bush et al. [18], 1 infant of 4 had a treated bleed during emicizumab prophylaxis (traumatic bleed before high-titer inhibitor diagnosis), and 1 infant of 5 in Wang et al. [60] had a shin hematoma at 23 months of age, which resolved after 1 dose of FVIII. In Barg et al. [61], none of the 5 infants experienced hemarthrosis or any spontaneous bleeds during 36 weeks of follow-up. Out of the 5 publications, only Barg et al. [32] included major safety events: major bleeding after circumcision in a 3-month-old (emicizumab plasma level was 31 μg/mL) treated by red blood cell transfusions, tranexamic acid, and recombinant activated FVII, and a fatal outcome in a 5-month-old who also presented with central venous line thrombosis, as previously described in section 3.3. There were 4 case reports/series that included infants initiating emicizumab. One infant with inhibitors (aged 6 months) had no breakthrough bleeding or AEs after almost 1 year of emicizumab treatment [62]. Two infants, aged ∼6 months and 12 months without inhibitors, started emicizumab and had no spontaneous bleeding or AEs for 9 and 5 months of follow-up, respectively [63,64]. A case series by Mason and Young [65] described 2 cases of infants who were <12 months commencing emicizumab: one started at 7 days old following initial treatment of intracranial hemorrhage with a FVIII infusion, and the other commenced at 5 weeks due to parental anxiety regarding the potential for intracranial hemorrhage during infancy. No bleeding or safety events were reported in these case reports, with a median follow-up of 12 months.

**Table 7:** Summary of publications focusing on populations of interest.

Setting	Author	Year	No. of patients	FVIII inhibitors	Safety data	Bleed data
PUPs and MTPs						
Clinical trials	Shima et al. [16]	2019	1 PUP	Without	No safety events reported	No bleeding events reported
Real-world studies	Bush et al. [18], Pompa et al. [66], Hassan et al. [58]	2020-2022	12 PUPs/MTPs	With and without	One case of injection-site bruising; no other safety events reported	Zero treated bleeds: range, 67%-100%; mean (SD), 89% (15.6); median, 100%
Case studies	Heine et al. [67]	2021	3 PUPs	With	N/A	One PUP had zero treated bleeds over 2 y of follow-up; data not reported for the other 2
Infants (≤12 mo)						
Clinical trials	Young et al. [57]	2019	8 (<2 y)	With	No safety data for infants, specifically ≤12 mo of age, were reported	N/A
	Shima et al. [16]	2019	1 PUP (4 mo)	Without	N/A	No treated bleeds
Real-world studies	Hassan et al. [58], Wieland et al. [59]	2022	9	With and without	N/A	Zero treated bleeds
	Wang et al. [60]	2022	5	With and without	N/A	One infant had a shin hematoma (23 mo) that resolved after 1 dose of FVIII
	Bush et al. [63]	2020	4	N/A	N/A	One infant had a treated bleed during emicizumab prophylaxis (traumatic bleed before a high-titer inhibitor diagnosis)
	Barg et al. [61]	2019	5	With	A major bleeding event was reported after circumcision in a 3-month-old (emicizumab plasma level, 31 μg/mL): treated by RBCT, TXA, and rFVIIa. One fatal outcome in a 5-month-old was reported	No cases of hemarthrosis or any spontaneous bleeds were reported during 36 wk of follow-up
Case studies	Campaniço et al. [64]	2022	1 (12 mo)	Without	No adverse events reported	No bleeding events reported
	Garcia et al. [62]	2021	1 (6 mo)	With	No adverse events reported	No bleeding events reported
	Mason and Young [65]	2021	2 (one 7 d of age and one 5 wk of age)	Without	No safety events reported (median follow-up of 12 mo)	No bleeding events reported (median follow-up of 12 mo)
	Bush et al. [63]	2020	1 (∼6 mo)	Without	No adverse events reported	No bleeding events reported
Older people with hemophilia A (≥50 y)						
Clinical trials	Jiménez-Yuste et al. [23]	2022	96	With and without	Safety data similar to the overall hemophilia A population (HAVEN 1, HAVEN 3, HAVEN 4, and STASEY)	ABRs similar to the overall hemophilia A population (HAVEN 1, HAVEN 3, HAVEN 4, and STASEY). Mean treated ABR (95% CI) was 1.39 (0.08-6.23) in the overall population and 1.82 (0.19-6.93)
Real-world studies	Lehtinen et al. [68]	2022	4	N/A	N/A	Three participants had zero treated bleeds, and 1 reported a traumatic breakthrough bleed
	Misgav et al. [20]	2021	6	With and without	No thrombotic events or thrombotic microangiopathies were reported	ABRs between 0.0 and 8.2 were reported (median value, 0.85)
	Peyvandi et al. [69]	2020	31 (≥65 y)	With and without	Nine fatalities were reported, none of which were related to emicizumab	N/A
Case studies	Weise et al. [70]	2022	1 (64 y)	N/A	Emicizumab was well tolerated	Patient was undergoing chronic intermittent hemodialysis. Emicizumab was efficacious; plasma levels remained stable throughout hemodialysis
Nonsevere hemophilia A						
Clinical trials	Hermans et al. [27]	2022	72	Without	No new safety signals, thrombotic events, or thrombotic microangiopathies were identified	Bleed data were consistent with the pivotal HAVEN studies (model-based ABR [95% CI], 0.8 [0.41-1.46])
Real-world studies	Buckner et al. [34]	2022	38	With and without	Safety profile consistent with previous analyses	ABRs were consistently low (mean [SD] ABR for treated bleeds: 1.3 [3.0])
	Poon et al. [71]	2021	73	With and without	No thrombotic events or thrombotic microangiopathies were observed	Zero bleeds of between 50% and 57% were reported; the median ABR ranged between 0 and 11 (1 participant experienced 11 bleeds; 8 were related to sporting injuries, and 3 were spontaneous)
Case studies	Camelo et al. [73]	2021	1	With	N/A	Patient had Melnick–Needles^a^ syndrome, and ABR reduced from 11 to 0 after 12 mo of treatment with emicizumab
	Rener et al. [72]	2020	1	With	No adverse events were reported	Patient had life-threatening hematuria. ABR of 0 after 12 mo of follow-up with emicizumab

ABR, annualized bleed rate; FVIII, factor VIII; MTP, minimally treated patient; PUP, previously untreated patient; RBCT, red blood cell transfusion; rFVIIa, recombinant activated factor VII; TXA, tranexamic acid.

a Melnick–Needles syndrome is a genetic disorder involving abnormalities in skeletal development.

#### 3.6.2 PUPs and MTPs

Data for PUPs and MTPs were scarce overall. Only 1 PUP was included in the clinical trial setting (a 4-month-old with severe hemophilia A as described in section 3.6.1) [16]. Collectively, 3 real-world studies presented data for 12 PUPs/MTPs [18,58,66] who initiated emicizumab between 1 and 23 months of age and were followed for 3 to 24 months. The percentages of participants with zero treated bleeds ranged from 67% to 100%; mean (SD) and median values were 89% (15.6) and 100%, respectively. Other than 1 case of injection-site bruising [18], no other safety events were highlighted for these 12 patients. One publication from Germany explored using emicizumab in PUPs (*n* = 3); the article reported data for 1 PUP (age at initiation of emicizumab not reported) who had no treated bleeds over 2 years of follow-up [67]. In one additional case report, the family of a 15-month-old PUP with severe hemophilia A elected to start emicizumab primarily to avoid venipuncture and central venous access device; at 28 months of age, the child had not experienced a bleed and was yet to receive FVIII concentrates [65].

#### 3.6.3 Older people with hemophilia A (aged ≥50 years)

While people with hemophilia A over 50 years of age were included in the emicizumab clinical trial program, only 1 publication, a post hoc analysis of HAVEN 1, HAVEN 3, HAVEN 4, and STASEY, reported data on this population [23]. The analysis examined the efficacy and safety of emicizumab in people with hemophilia A aged ≥50 years with cardiovascular risk factors, HIV, and prior/current hepatitis C virus infection, with reported ABRs and safety data similar to the overall population of the 4 trials. Two real-world studies presented bleed data on 10 people with hemophilia A (≥50 years). Lehtinen et al. [68] included 4 participants aged ≥50 years; 3 had zero treated bleeds after 3 months of maintenance emicizumab, and 1 had a traumatic breakthrough bleed, which resolved quickly with 1 to 2 doses of extended half-life FVIII. In Misgav et al. [20], 6 elderly people with hemophilia A with comorbidities (multiple cardiovascular risk factors) had ABRs ranging from 0.0 to 8.2 (median, 0.85); neither thrombotic events nor thrombotic microangiopathy were reported. There was also an Emicizumab Global Safety Database report (not included in the overall analysis due to overlap with other publications), which investigated fatal events contemporaneous to emicizumab and included 31 individuals over the age of 65 [69]. The report included 9 fatalities; however, none were reported as related to emicizumab. One case report described the use of emicizumab in a 64-year-old male with severe hemophilia A undergoing chronic intermittent hemodialysis; emicizumab was well tolerated and efficacious, and plasma levels remained stable throughout hemodialysis [70].

#### 3.6.4 Non-severe hemophilia A

The primary analysis results for the phase 3 HAVEN 6 clinical trial were captured, showing data for those with non-severe hemophilia A on emicizumab [27]. Efficacy data were consistent with other HAVEN studies (model-based ABR [95% CI], 0.8 [0.41-1.46]), and no new safety signals, thrombotic events, or thrombotic microangiopathy were identified. Two real-world studies [34,71] also reported data for people with mild or moderate hemophilia A. ATHN 7, a natural history cohort study of the safety, effectiveness, and practice of treatment for people with hemophilia, included 38 people with mild or moderate hemophilia A treated with emicizumab; 6 of these individuals had inhibitors against FVIII [34]. ABRs were consistently low for participants with non-severe hemophilia A (mean [SD] ABR for treated bleeds, 1.3 [3.0]), and the safety profile was consistent with previous analyses. Data from the Canadian Bleeding Disorders Registry reported a range of zero bleeds between 50% and 57%, and the median ABR ranged between 0 and 11 (1 patient experienced 11 bleeds; 8 related to sporting injuries, and 3 spontaneous) [71]. No thrombotic events or thrombotic microangiopathy were observed. Two additional case reports described the use of emicizumab in people with mild hemophilia A with high-titer inhibitors. The first was in a patient with life-threatening hematuria, where emicizumab therapy led to resolution after 1 week; after 12 months of followup, the patient had an ABR of 0 with no AEs [72]. The second was in a woman with Melnick–Needles syndrome, a genetic disorder involving abnormalities in skeletal development, whose ABR was reduced from 11 to 0 after 12 months of treatment with emicizumab [73].

## 4 Discussion

To the best of our knowledge, this scoping review is the first to bring together the published global experience with emicizumab prophylaxis as a treatment for congenital hemophilia A from both the clinical and real-world settings since its initial approval in 2017. Collectively, results demonstrate that emicizumab is a well-tolerated and effective prophylaxis option that reduces bleeding events while alleviating the burden of intravenous infusions associated with FVIII replacement therapies. Emicizumab has facilitated the acceptance of prophylaxis as the new global standard of care, which has shaped the management of hemophilia A in people with or without inhibitors to FVIII and how they lead their lives [74]. With emicizumab, people with hemophilia A now have an effective and tolerable subcutaneous treatment option, offering a stable level of bleed prevention over time, in contrast to the peaks and troughs of FVIII replacement therapy, and has the added benefit of less burdensome treatment regimens and improved QoL. Findings from this scoping review further confirm that emicizumab is a valuable option in all people with congenital hemophilia A, including people of all ages and severities with a risk of bleeding, with or without FVIII inhibitors, and those who struggle with intravenous administration [75].

### 4.1 Bleed outcomes

Overall, the numbers of bleeds were low and aligned across settings; calculated mean and median ABRs for treated bleeds across all studies were <1 (0.6 and 0.0, respectively), and the mean percentage of people with zero treated bleeds was 65.0% (observation time ranging between ∼19 weeks and ∼2.4 years). Due to different endpoints being reported between studies, not all studies/publications were used for each type of outcome, and the between-study variability in observation times (ie, ∼19 weeks to ∼2.4 years) made it difficult to consolidate and compare results.

While the primary analyses efficacy data from the individual clinical trial publications were used, the longer-term pooled data from Callaghan et al. [13] are also supportive of the ongoing bleed prevention and tolerability of emicizumab prophylaxis. Additionally, efficacy findings are consistent with a number of publications that have become available since the cut-off date of August 9, 2022 [15,24,[76], [77], [78], [79], [80], [81]].

### 4.2 Safety outcomes

These collective data confirm the safety profile and tolerability of emicizumab prophylaxis in people with hemophilia A. Still, some differences were apparent in the ranges reported for safety outcomes in clinical trials versus real-world publications, eg, the percentage of the population who had ≥1 AE ranged from 70.4% to 100% in clinical trials and 0.0% to 70.6% in real-world studies. These variable ranges could be explained by several factors, including differences in safety reporting, ie, less rigorous criteria, reliance on self-reporting AEs in real-world studies, and both clinicians and patients becoming more comfortable with emicizumab administration over time. Of note, the most common treatment-related AEs overall were ISRs (observed in 0.0%-60.0% of participants across clinical and real-world settings; median, 10.2%; *n* = 1607).

Across 41 publications describing >2300 people with hemophilia A who received emicizumab prophylaxis, the total number of thrombotic events and thrombotic microangiopathy events was 11 and 4, respectively. In a recent summary published after the cut-off date [82], a total of 57 non–APCC-associated thrombotic events were reported since the first use of emicizumab. The higher number of thrombotic events reported in Koparkar et al. [82] compared with this review (57 vs 11, respectively) is due to the additional inclusion of spontaneous reports from preapproval programs, ie, compassionate use in people with inhibitors to FVIII (who had exhausted available treatment options and were often in life-threatening condition), and the postmarketing setting; there is also a risk of duplicate reports across these settings. After an increased risk of thrombotic events and thrombotic microangiopathy observed in HAVEN 1, when emicizumab was used in conjunction with high doses of APCC, guidance to avoid use of emicizumab with APCC >100 U/kg/24 h for ≥24 hours was introduced; there have since been no new cases of thrombotic microangiopathy in the clinical trial setting. Eight fatalities were identified in 41 publications (*n* = 2246); no fatalities were assessed as related to emicizumab, which was also the case with another publication that examined safety events, including fatalities, from the Emicizumab Global Safety Database [69]. The latter publication was excluded from our analysis as it included aggregated data that would have duplicated events and fatalities already included via other published reports.

The incidence of ADAs with or without loss of efficacy remained low, unlike FVIII inhibitor development, which exists at ∼30% with FVIII replacement therapies. Additional matters, such as the risk of recurrence of FVIII inhibitors following exposure to emicizumab, will be addressed with future studies and further real-world evidence.

Case studies reporting safety events for people with hemophilia A receiving emicizumab have largely described AEs unrelated to emicizumab. With the exception of ADA development and hematuria, all emicizumab-related events were successfully resolved. The majority of cases reporting ADAs were in people with FVIII inhibitors [43,44,46,47]; however, cases have also been reported in those without FVIII inhibitors [45,83].

### 4.3 QoL

Data from well-established tools such as the Haem-A-QoL, Haemophilia QoL, and EQ-5D-5L support the QoL benefits of emicizumab in clinical trials [10,14,[48], [49], [50], [51], [52]]. While these tools are not typically used in the real-world setting, there are qualitative reports in the literature that support the improved QoL, both physically and mentally, experienced by people with hemophilia A taking emicizumab [84,85]. Additional data are warranted to evaluate the QoL benefits of emicizumab in populations for which few results currently exist. Many of these QoL assessment tools were developed to measure outcomes with FVIII replacement therapies rather than emicizumab and do not take into account the disability paradox, where people with hemophilia can underestimate the burden of their disease in comparison with the general population [86].

### 4.4 Subgroup analyses

Historically, clinical trials have focused on typically fit children and adults (aged ∼2-65 years) with severe hemophilia A. Owing to the specific needs of various subpopulations, such as infants and older people with hemophilia A, it becomes necessary to gain further insights into the treatment of these patients as well as the original broader population. While patient subgroups, such as infants ≤12 months and people with nonsevere hemophilia, are often listed as part of broader data sets, very few report on them in detail. Clinical and real-world evidence so far supports the safety profile and efficacious bleed prevention of emicizumab in people with hemophilia A regardless of disease severity or age, with the youngest patient being treated at 7 days old and the oldest over 70 years old [20,65]. Moreover, clinical trials of emicizumab use in specific populations of interest are emerging, aiming to provide further evidence of its safety and stable prevention against bleeds. For example, the HAVEN 7 clinical trial primary analysis (NCT04431726; published after the cut-off date) showed that over 52 weeks, emicizumab was efficacious and well tolerated in 55 infants with severe hemophilia A without FVIII inhibitors (median [range] treatment duration, 100.3 [52-118] weeks); 54.5% of participants had zero treated bleeds and no deaths, intracranial hemorrhages, thrombotic events, or thrombotic microangiopathy were reported [22]. HAVEN 7 includes a 7-year follow-up to describe the impact of initiating prophylaxis with emicizumab as early as 9 days after birth, and long-term outcomes such as joint health in this population. Additionally, the HAVEN 6 clinical trial was the first to look at emicizumab in people with moderate and mild hemophilia A, who are historically an underserved population [24]. This led to the European Medicines Agency label expansion of emicizumab for people with moderate disease who have a severe bleeding phenotype (February 2023). This has now prompted interest in further understanding the real-world experience of emicizumab in this non-severe population, including women with hemophilia A [73,87]. It is expected that more real-world data will start to emerge in these subpopulations over the next few years.

### 4.5 Limitations

Owing to the broadness of the data captured, no comprehensive statistical analysis was performed. Inconsistencies in the reporting of bleed outcomes, ie, no specification on types of bleeds and reporting per arm in clinical trials versus the overall population in real-world studies, are a limitation of this scoping review and make it challenging to perform any detailed statistical analysis. This further makes the case for standardized outcome reporting within and between clinical trials and real-world studies so that data can be collated and compared effectively. While every effort was made to ensure that the same patient or population was not captured more than once, there is a potential risk that some individuals were included in both multinational and national registries as well as separate country studies.

This scoping review focuses solely on congenital hemophilia A and does not consider emicizumab in other indications, such as acquired hemophilia A and von Willebrand disease. Additionally, due to the few publications reporting on women and registries often not specifying whether women were included, this limits the conclusions we can make for the use of emicizumab in women with hemophilia A. While this scoping review does not cover all aspects of patient management, ie, joint health and surgery, additional data are available and continually emerging to address these knowledge gaps [78,[88], [89], [90]].

## 5 Conclusions

Despite the differences in data capture and outputs across clinical trials and real-world settings, this scoping review is the first to consolidate the published global experience and data for emicizumab in people with congenital hemophilia A. In line with the pivotal study outcomes that enabled the approval of emicizumab 6 years ago, these data support its acceptable tolerability, effectiveness, and positive impact on QoL, regardless of age, disease severity, or FVIII inhibitor status.
