# Supplementary material for: Emicizumab is well tolerated and effective in people with congenital hemophilia A regardless of age, severity of disease, or inhibitor status: a scoping review
Source: Res Pract Thromb Haemost. 2024 Apr 18;8(4):102415. doi: 10.1016/j.rpth.2024.102415 (PMC11135026; doi:10.1016/j.rpth.2024.102415)
Supplement: Supplementary Material [file mmc1.docx]

**SUPPLEMENTARY MATERIAL**

**TABLE S1.** Preferred Reporting Items for Systematic reviews and Meta-Analyses extension for Scoping Reviews (PRISMA-ScR) Checklist.

| **SECTION** | **ITEM** | **PRISMA-ScR CHECKLIST ITEM** | **REPORTED ON PAGE #** |
| --- | --- | --- | --- |
| **TITLE** | | | |
| Title | 1 | Identify the report as a scoping review. | Page 1 |
| **ABSTRACT** | | | |
| Structured summary | 2 | Provide a structured summary that includes (as applicable): background, objectives, eligibility criteria, sources of evidence, charting methods, results, and conclusions that relate to the review questions and objectives. | Page 3 |
| **INTRODUCTION** | | | |
| Rationale | 3 | Describe the rationale for the review in the context of what is already known. Explain why the review questions/objectives lend themselves to a scoping review approach. | Pages 5 and 6 |
| Objectives | 4 | Provide an explicit statement of the questions and objectives being addressed with reference to their key elements (e.g., population or participants, concepts, and context) or other relevant key elements used to conceptualize the review questions and/or objectives. | Pages 7, 8 and 9  Table 1  Table 2 |
| **METHODS** | | | |
| Protocol and registration | 5 | Indicate whether a review protocol exists; state if and where it can be accessed (e.g., a Web address); and if available, provide registration information, including the registration number. | Page 27 |
| Eligibility criteria | 6 | Specify characteristics of the sources of evidence used as eligibility criteria (e.g., years considered, language, and publication status), and provide a rationale. | Pages 7 and 8 |
| Information sources* | 7 | Describe all information sources in the search (e.g., databases with dates of coverage and contact with authors to identify additional sources), as well as the date the most recent search was executed. | Page 7  Table S3 |
| Search | 8 | Present the full electronic search strategy for at least 1 database, including any limits used, such that it could be repeated. | Table S2 |
| Selection of sources of evidence^†^ | 9 | State the process for selecting sources of evidence (i.e., screening and eligibility) included in the scoping review. | Pages 7 and 8  Table 1 |
| Data charting process^‡^ | 10 | Describe the methods of charting data from the included sources of evidence (e.g., calibrated forms or forms that have been tested by the team before their use, and whether data charting was done independently or in duplicate) and any processes for obtaining and confirming data from investigators. | Page 8 |
| Data items | 11 | List and define all variables for which data were sought and any assumptions and simplifications made. | Pages 8 and 9 |
| Critical appraisal of individual sources of evidence^§^ | 12 | If done, provide a rationale for conducting a critical appraisal of included sources of evidence; describe the methods used and how this information was used in any data synthesis (if appropriate). |  |
| Synthesis of results | 13 | Describe the methods of handling and summarizing the data that were charted. | Pages 8 and 9 |
| **RESULTS** | | | |
| Selection of sources of evidence | 14 | Give numbers of sources of evidence screened, assessed for eligibility, and included in the review, with reasons for exclusions at each stage, ideally using a flow diagram. | Page 10  Table 3  Figure 1 |
| Characteristics of sources of evidence | 15 | For each source of evidence, present characteristics for which data were charted and provide the citations. | Table 7  Table S4  Table S5  Table S6  Table S7  Table S8  Table S9 |
| Critical appraisal within sources of evidence | 16 | If done, present data on critical appraisal of included sources of evidence (see item 12). |  |
| Results of individual sources of evidence | 17 | For each included source of evidence, present the relevant data that were charted that relate to the review questions and objectives. | Pages 10–18 |
| Synthesis of results | 18 | Summarize and/or present the charting results as they relate to the review questions and objectives. | Table 4  Table 5  Table 6  Table 7  Figure 2 |
| **DISCUSSION** | | | |
| Summary of evidence | 19 | Summarize the main results (including an overview of concepts, themes, and types of evidence available), link to the review questions and objectives, and consider the relevance to key groups. | Pages 19–23 |
| Limitations | 20 | Discuss the limitations of the scoping review process. | Pages 22 and 23 |
| Conclusions | 21 | Provide a general interpretation of the results with respect to the review questions and objectives, as well as potential implications and/or next steps. | Page 24 |
| **FUNDING** | | | |
| Funding | 22 | Describe sources of funding for the included sources of evidence, as well as sources of funding for the scoping review. Describe the role of the funders of the scoping review. | Page 27 |

^*^Where *sources of evidence* (see second footnote) are compiled from, such as bibliographic databases, social media platforms, and Web sites.

^†^A more inclusive/heterogeneous term used to account for the different types of evidence or data sources (e.g., quantitative and/or qualitative research, expert opinion, and policy documents) that may be eligible in a scoping review as opposed to only studies. This is not to be confused with *information sources* (see first footnote).

^‡^ The frameworks by Arksey and O’Malley (6) and Levac and colleagues (7) and the JBI guidance (4, 5) refer to the process of data extraction in a scoping review as data charting*.*

^§^ The process of systematically examining research evidence to assess its validity, results, and relevance before using it to inform a decision. This term is used for items 12 and 19 instead of “risk of bias” (which is more applicable to systematic reviews of interventions) to include and acknowledge the various sources of evidence that may be used in a scoping review (e.g., quantitative and/or qualitative research, expert opinion, and policy document). Abbreviations: JBI, Joanna Briggs Institute; PRISMA-ScR, Preferred Reporting Items for Systematic reviews and Meta-Analyses extension for Scoping Reviews.

From: Tricco AC, Lillie E, Zarin W, O’Brien KK, Colquhoun H, Levac D, et al. PRISMA Extension for Scoping Reviews (PRISMAScR): Checklist and Explanation. Ann Intern Med. 2018;169:467–473. [doi: 10.7326/M18-0850](http://annals.org/aim/fullarticle/2700389/prisma-extension-scoping-reviews-prisma-scr-checklist-explanation)

**TABLE S2**. Search terms

| **Search number** | **Query** | **Subject** |
| --- | --- | --- |
| 1 | “emicizumab” [Supplementary Concept] OR Hemlibra[all] OR ACE910[all] OR “ACE-910”[all] OR “emicizumab-kxwh”[all] OR emicizumab[all] **OR bispecific monoclonal antibody OR FVIII mimetic** | **Emicizumab** |
| 2 | (“Hemophilia A”[Mesh] OR “Hemophilia A”[tiab] OR “Hemophilia”[tiab] OR “Congenital Hemophilia A”[tiab] OR “Classic Hemophilia”[tiab] OR “Autosomal Hemophilia A”[tiab] OR “Factor VIII Deficiency”[tiab] OR “Factor 8 Deficiency”[tiab] OR “Congenital Factor VIII Deficiency”[tiab] OR “Haemophilia A"[“iab] OR "Congenital Haemophilia A”[tiab] OR “Classic Haemophilia”[tiab] OR “Autosomal Haemophilia A”[tiab]) NOT (“hemophilia b”[ti] OR hemophiliacs[tiab] OR “hemophiliac patient*”[tiab]) | **Hemophilia A** |
| 3 | #1 AND #2 | **Emi + HemA** |
| 4 | “Review”[Publication Type] OR “Editorial”[Publication Type] OR “biography”[Publication Type] OR “comment”[Publication Type] OR “editorial”[tiab] | **Publication types to exclude** |
| 5 | #3 NOT #4 | **Unwanted pub types excluded** |
| 6 | #5 NOT (“animals”[Mesh] NOT “humans”[Mesh]) | **Restrict to human subjects** |
| 7 | #6 NOT (“In Vitro Techniques”[Mesh] OR “in vitro”[ti]) | **Exclude in vitro studies** |
| 8 | #7 AND eng[la] | **Restrict to English language only** |
| 9 | #8 AND (2015/1/1:3000/12/12[pdat]) | **Restrict to publication after 2014** |

**TABLE S3.** Manually searched congress abstract books.

| **Congress** |
| --- |
| European Association for Haemophilia and Allied Disorders (EAHAD) |
| International Society on Thrombosis and Haemostasis (ISTH) |
| American Society of Hematology (ASH) |
| World Federation of Hemophilia (WFH) |
| European Hematology Association (EHA) |
| National Hemophilia Foundation (NHF) |
| Hemophilia Federation of America (HFA) |

**TABLE S4.** Characteristics of clinical trials included for bleed analysis.

| **Author, year** | **Study name** | **Population** | **Dose of emicizumab** | **Sample size** | **Zero-bleed outcomes, %** | | **ABR outcomes** | | |
| --- | --- | --- | --- | --- | --- | --- | --- | --- | --- |
|  |  |  |  |  | **Treated** | **All bleeds** | **Model based** | **Calculated mean** | **Calculated median** |
| Hermans 2022 | HAVEN 6 | Moderate/mild hemophilia A without FVIII inhibitors  Pediatric, adolescent and adult patients | QW, Q2W, Q4W | 72 | 66.7 | 33.3 | 0.9 | 0.9 | 0.0 |
| Yang 2022 | HAVEN 5 | Adults and adolescents (≥12 years) with severe hemophilia A without FVIII inhibitors or hemophilia A of any severity with FVIII inhibitors | QW | 29 | 65.5 | 33.3 | 1.0 | - | - |
|  |  |  | Q4W | 27 | 55.6 | 37.9 | 1.0 | - | - |
| Jiménez-Yuste 2021 | STASEY | People ≥12 years old with FVIII inhibitors | QW | 195 | 82.6 | 54.9 | 0.5 | - | 0 |
| Shima 2019 | HOHOEMI | Pediatric patients (<12 years) with severe hemophilia A without FVIII inhibitors | Q2W | 6 | 33.3 | 0 | 1.3 | 1.3 | 1.4 |
|  |  |  | Q4W | 7 | 71.4 | 14.3 | 0.7 | 0.7 | 0 |
| Pipe 2019 | HAVEN 4 | People with hemophilia A with or without FVIII inhibitors | Q4W | 41 | 56.1 | 29.3 | 2.4 | - | 0 |
| Young 2019 | HAVEN 2 | Pediatric patients (<12 years) with severe hemophilia A with FVIII inhibitors | QW | 65 | 76.9 | 49.2 | 0.3 | - | 0 |
| Young 2022 |  |  | Q2W | 10 | 70.0 | 40.0 | 0.2 | - | - |
|  |  |  | QW4 | 10 | 60.0 | 30.0 | 1.8 | - | - |
| Mahlangu 2018 | HAVEN 3 | People with hemophilia A without FVIII inhibitors | QW | 36 | 56.0 | 50.0 | 1.5 | - | 0 |
|  |  |  | Q2W | 35 | 60.0 | 40.0 | 1.3 | - | 0 |
|  |  |  | QW (prior prophy) | 63 | 55.6 | 44.4 | 1.6 | - | 0 |
| Oldenburg 2017 | HAVEN 1 | People with hemophilia A with FVIII inhibitors | QW (prev. epi BPA) | 35 | 62.9 | 37.1 | 2.9 | - | 0 |
|  |  |  | QW (prev. prophy BPA) | 49 | 69.4 | - | 5.1 | - | 0 |

While every effort was made to ensure that the same patient was not captured more than once in these data, there is a potential risk that some individuals were included in more than one publication. Abbreviations: ABR, annualized bleed rate; FVIII, factor VIII; QW, once weekly; Q2W, every 2 weeks; Q4W, every 4 weeks.

**TABLE S5.** Characteristics of real-world studies included for bleed analysis.

| **Study** | **Population** | | **Dose of emicizumab** | **Sample size** | **Zero-bleed outcomes, %** | | | **ABR outcomes** | |
| --- | --- | --- | --- | --- | --- | --- | --- | --- | --- |
|  |  |  |  |  | **Spontaneous** | **Treated** | **All bleeds** | **Calculated mean** | **Calculated median** |
| Abdelwahab 2022 | Pediatric and adolescent patients with severe hemophilia A and FVIII inhibitors | | Q2W | 14 | - | - | 78.6 | - | - |
| Arcudi 2022 | Pediatric and adult patients with severe hemophilia A with and without inhibitors | | QW, Q2W | 21 | - | - | 71.7 | - | - |
| Buckner 2022 | Patients with hemophilia A of all severities without FVIII inhibitors | | - | 188 | - | - | - | 1.35 | 0.42 |
| Fletcher 2022 | Pediatric and adult patients (8–63 years) with FVIII inhibitors | | - | 15 | - | - | 60.0 | - | - |
| Harroche 2022 | Patients of all ages (<2 years) and all severities of hemophilia A | | - | 119 | - | - | 76.5 | 0.23 | - |
| Hassan 2022 | Infants with severe hemophilia A (PTPs and MTPs) | | Q2W | 5 | - | - | 100.0 | - | - |
| Hassan and Motwani 2022 | Pediatric patients with severe hemophilia A with and without FVIII inhibitors  PTPs, PUPs and MTPs | | QW, Q2W | 51 | - | - | 56.8 | - | - |
| Mondorf 2022 | Severe hemophilia A | | - | 26 | - | - | 77.0 | - | - |
| Nakajima 2022 | Patients with mild or severe hemophilia A | | - | 63 | - | - | 69.8 | - | - |
| Taal 2022 | Severe hemophilia A | | - | 119 | - | - | 77.0 | - | - |
| Wall 2022 | Severe hemophilia A without FVIII inhibitors | | - | 259 | - | - | 69.9 | - | - |
| Wang 2022 | Infants with severe hemophilia A | | - | 6 | - | - | 83.3 | - | - |
| Wieland 2022 | Infants (3–26 months) with moderate or severe hemophilia A | | - | 4 | 100.0 | - | - | - | - |
| Al-Samkari 2021 | Pediatric and adult (0–75) patients | With FVIII inhibitors | - | 15 | - | - | - | - | 0 |
|  |  | Without FVIII inhibitors | - | 34 | - | - | - | - | 0 |
| Arcudi 2021 | Pediatric (PUPs and PTPs) and adult patients with severe or moderate hemophilia A, with and without FVIII inhibitors | | - | 22 | - | - | 72.7 | - | - |
| Barg 2021 | Severe hemophilia A with and without inhibitors | Adult | - | 107 | - | - | 49.5 | - | 0.5 |
|  |  | Pediatric |  |  |  |  |  |  | 0.25 |
| Batsuli 2021 | Pediatric patients with severe hemophilia A with a history of, or current, FVIII inhibitors | | QW, Q2W | 12 | - | - | 50.0 | - | - |
| Batt 2021 | Pediatric and adult (2–63 years) patients without FVIII inhibitors | | - | 121 | - | - | - | 0.55 | - |
| Campaniço 2021 | Pediatric and adult patients with FVIII inhibitors | | - | 9 | - | 77.8 | - | - | - |
| Chuansumrit 2021 | Pediatric and adult (4–40 years) patients with moderate or severe hemophilia A with and without FVIII inhibitors | | 1.5–6 mg/kg QW–Q4W | 6 | - | - | 66.7 | - | - |
| Cohen 2021 | Pediatric patients (≤18 years) with and without FVIII inhibitors | | Q2W | 28 | - | - | 67.9 | - | - |
| Hassan and Motwani 2021 | Pediatric patients with and without FVIII inhibitors | | - | 49 | - | 85.7 | - | 0.29 | 0 |
| Hassan, Jonathan and Jayashree 2021 | Pediatric patients (1–16.7 years) with severe hemophilia A with and without FVIII inhibitors | | QW, Q2W, Q4W | 42 | - | - | 80.7 | - | - |
| Kenet 2021 | Pediatric patients with hemophilia A of all severities with and without FVIII inhibitors | | - | 141 | - | - | 58.0 | - | - |
| Khairnar 2021 | Pediatric and adult patients with hemophilia A of all severities with and without FVIII inhibitors | | - | 19 | - | - | 79.0 | - | - |
| Levy-Mendelovich 2021 | Pediatric and adult patients (1 month–74.6 years) with severe hemophilia A | | QW, Q2W | 70 | 48.6 | - | - | - | - |
| Misgav 2021 | Adult (>50 years) patients with and without cardiovascular risk factors | With FVIII inhibitors | - | 4 | - | - | 29.4 | - | 0.5 |
|  |  | Without FVIII inhibitors | - | 13 | - | - |  | - | 1 |
| Nunez Toscano 2021 | Patients with severe hemophilia A with FVIII inhibitors | | - | 7 | - | - | 57.1 | - | - |
| Panovska-Stavridis 2021 | Adolescent and adult patients (16–62 years) with severe hemophilia A without FVIII inhibitors | | - | 10 | - | 70.0 | - | - | - |
| PompaGarza 2021 | Pediatric and adult patients with severe hemophilia A | | QW, Q2W, Q4W | 10 | - | - | 80.0 | - | - |
| Poon 2021 | Pediatric and adult patients with and without FVIII inhibitors | | QW, Q2W, Other | 73 | - | - | 80.8 | - | - |
| Pouplard 2021 | Pediatric and adult patients with severe hemophilia A with and without inhibitors | | - | 183 | - | - | 88.0 | - | - |
| Radhakrisjnan 2021 | Severe hemophilia A with FVIII inhibitors | | QW, Q2W, Q4W, 2–3 mg/kg Q2W–Q6W | 7 | 100.0 | - | - | - | - |
| Riera 2021 | Adult patients (20–48 years) with and without inhibitors | | - | 5 | - | - | 60.0 | - | - |
| Schiavulli 2021 | Pediatric patients with severe hemophilia A with and without FVIII inhibitors | | - | 18 | - | - | 100.0 | - | - |
| Warren 2021 | Pediatric and adult patients (0.55–79.8 years) with hemophilia A of all severities with previous or current FVIII inhibitors | | QW, Q2W, Q4W, Other | 68 | - | - | 36.8 | - | - |
| Windyga 2021 | Adult patients (23–70 years) with FVIII inhibitors | | - | 20 | - | - | 90.0 | - | - |
| Barg 2020 | Pediatric patients with severe hemophilia A with and without FVIII inhibitors | | QW | 40 | - | - | 50.0 | - | - |
| Bush 2020 | Infants (1–23 months), PUPs and MTPs with severe hemophilia A | | QW, Q4W | 6 | - | 66.7 | - | - | - |
| Catarino 2020 | Pediatric (4 months–12 years) and adult (27–55 years) patients with FVIII inhibitors | | - | 17 | - | 71.0 | - | - | - |
| Ebbert 2020 | Pediatric and adult patients with hemophilia A of all severities with and without FVIII inhibitors | | - | 42 | - | - | 67.7 | - | - |
| Garcia 2020 | Pediatric patients with severe hemophilia A with and without inhibitors | | QW, Q2W | 30 | - | - | 83.3 | - | - |
| Lewandowska 2020 | Pediatric and adult (1–66 years) patients with moderate or severe hemophilia A with and without FVIII inhibitors | | - | 122 | - | 50.8 | - | - | - |
| Ling 2020 | Patients with severe hemophilia A with and without inhibitors | | - | 21 | - | - | 90.5 | - | - |
| McCary 2020 | Pediatric patients (<12 years) | With FVIII inhibitors | QW, Q2W, Q4W | 19 | - | - | 90.3 | 0.4 | 0 |
|  |  | Without FVIII inhibitors |  | 74 | - | - |  | 0.4 | 0 |
| OngTang 2020 | Patients with severe hemophilia A with FVIII inhibitors | | - | 8 | - | - | 87.5 | - | - |
| Batsuli 2019 | Pediatric patients (21 months–12 years) with severe hemophilia A with FVIII inhibitors | | - | 7 | - | - | 42.8 | - | - |

While every effort was made to ensure that the same patient was not captured more than once in these data, there is a potential risk that some individuals were included in more than one publication. Abbreviations: ABR, annualized bleed rate; FVIII, factor VIII; MTP, minimally treated patient; QW, once weekly; Q2W, every 2 weeks; Q4W, every 4 weeks; Q6W, every 6 weeks; PUP, previously untreated patient.

**TABLE S6.** Characteristics of clinical trials included for safety analysis

| **Author, year** | **Study name** | **Population** | **Dose of emicizumab** | **Sample size** | **Participants (%)** | | | | | | |
| --- | --- | --- | --- | --- | --- | --- | --- | --- | --- | --- | --- |
|  |  |  |  |  | **≥1 AE** | **≥1 SAE** | **≥1 TRAE** | **≥1 ISR** | **≥1 TE** | **≥1 TMA** | **Death** |
| Hermans 2022 | HAVEN 6 | Moderate/mild hemophilia A without FVIII inhibitors  Pediatric, adolescent and adult patients | QW, Q2W, Q4W | 72 | 83.3 | 11.1 | 20.8 | 16.7 | 1.4 | 0.0 | 0.0 |
| Yang 2022 | HAVEN 5 | Adults and adolescents (≥12 years) with severe hemophilia A without FVIII inhibitors or hemophilia A of any severity with FVIII inhibitors | QW | 29 | 86.2 | 6.9 | 41.4 | 13.8 | 0.0 | 0.0 | 0.0 |
|  |  |  | Q4W | 27 | 70.4 | 3.7 | 37.0 | 18.5 | 0.0 | 0.0 | 0.0 |
| Jiménez-Yuste 2021 | STASEY | People ≥12 years old with FVIII inhibitors | QW | 193 | 84.5 | 16.1 | 18.1 | 9.8 | 1.04 | 0.0 | 1.04† |
| Shima 2019 | HOHOEMI | Pediatric patients (<12 years) with severe hemophilia A without FVIII inhibitors | Q2W | 6 | 100 | 16.7 | 16.7 | 16.7 | 0.0 | 0.0 | 0.0 |
|  |  |  | Q4W | 7 | 100 | 14.3 | 0.0 | 0.0 | 0.0 | 0.0 | 0.0 |
| Pipe 2019 | HAVEN 4 | People with hemophilia A with or without FVIII inhibitors | Q4W | 41.0 | 73.0 | 2.0 | 29.0 | 22.0 | 0.0 | 0.0 | 0.0 |
| Young 2019 | HAVEN 2 | Pediatric patients (<12 years) with severe hemophilia A with FVIII inhibitors | QW | 96.2 | 20.6 | 32.4 | 27.9 | 0.0 | 0.0 | 0.0 | 0.0 |
| Young 2022 |  | Pediatric patients (<12 years) with severe hemophilia A with FVIII inhibitors | Q2W | 10 | 90.0 | 10.0 | 20.0 | 20.0 | 0.0 | 0.0 | 0.0 |
|  |  |  | QW4 | 10 | 100.0 | 30.0 | 60.0 | 60.0 | 0.0 | 0.0 | 0.0 |
| Mahlangu 2018 | HAVEN 3 | People with hemophilia A without FVIII inhibitors | QW | 36 | 94.4 | 2.8 | 25.0 | 25.0 | 0.0 | 0.0 | 0.0 |
|  |  |  | Q2W | 35 | 85.7 | 8.6 | 31.4 | 20.0 | 0.0 | 0.0 | 0.0 |
|  |  |  | QW (prior prophy) | 63 | 87.3 | 15.9 | 38.1 | 32.0 | 0.0 | 0.0 | 0.0 |
| Oldenburg 2017 | HAVEN 1 | People with hemophilia A with FVIII inhibitors | QW (prev. epi BPA) | 34 | 85.3 | 11.8 | 38.2 | 23.5 | 2.9 | 2.9 | 2.8* |
|  |  |  | QW (prev. prophy BPA) | 49 | 71.4 | 8.2 | 18.4 | 10.2 | 2.0 | 2.0 | 0.0 |

While every effort was made to ensure that the same patient was not captured more than once in these data, there is a potential risk that some individuals were included in more than one publication

*One patient died due to rectal hemorrhage. ^†^One patient died because of polytrauma with a fatal head injury and another patient died due to abdominal compartment syndrome. Abbreviations: AE, adverse event; FVIII, factor VIII; ISR, injection-site reaction; SAE, serious adverse event; TE, thrombotic event; TMA, thrombotic microangiopathy; TRAE, treatment-related adverse event.

**TABLE S7.** Characteristics of real-world studies included for safety analysis.

| **Study** | **Population** | **Dose of emicizumab** | **Sample size** | **Participants (%)** | | | | | | | | |
| --- | --- | --- | --- | --- | --- | --- | --- | --- | --- | --- | --- | --- |
|  |  |  |  | **≥1 AE** | **≥1 SAE** | **≥1 TRAE** | **≥1 LISR** | **≥1 TE** | **≥1 TMA** | **Death** | **ADAs** | **ADAs and loss of efficacy** |
| Buckner 2022 | Patients with and without FVIII inhibitors | - | 253 | 3.6 | - | 1.2 | 1.2 | 0.0 | 0.0 | 0.4* | - | - |
| Hassan 2022 | Infants with severe hemophilia A (PTPs and MTPs) | Q2W | 5 | 0.0 | 0.0 | 0.0 | 0.0 | 0.0 | 0.0 | 0.0 | - | - |
| Hassan and Motwani 2022 | Pediatric patients with severe hemophilia A with and without FVIII inhibitors  PTPs, PUPs and MTPs | QW, Q2W | 51 | 0.0 | 0.0 | 0.0 | 0.0 | 0.0 | 0.0 | - | - | - |
| Rener 2022 | Pediatric and adult patients (7–57 years) with and without FVIII inhibitors | - | 6 | - | - | - | - | 0.0 | 0.0 | - | - | - |
| Taal 2022 | Severe hemophilia A | - | 119 | 1.85 | - | - | - | - | - | - | - | - |
| Wall 2022 | Patients with severe hemophilia A without FVIII inhibitors | - | 673 | - | - | - | - | - | - | - | 0.15 | - |
| Arcudi 2021 | Pediatric (PUPs and PTPs) and adult patients with severe or moderate hemophilia A, with and without FVIII inhibitors | - | 22 | - | - | - | - | 0.0 | 0.0 | - | 9.1 | 4.5 |
| Barg 2021 | Severe hemophilia A with and without inhibitors | - | 107 | 0.93 | - | 13.0 | 10.0 | 0.9† | 0.0 | 0.9‡ | 0.9 | - |
| Batsuli 2021 | Pediatric patients with severe hemophilia A with a history of, or current, FVIII inhibitors | QW, Q2W | 12 | - | - | - | - | - | - | 0.0 | - | - |
| Batsuli 2021 | Pediatric patients (4–14 years) with a history of FVIII inhibitors | - | 7 | - | - | - | - | 0.0 | 0.0 | 0.0 | - | - |
| Campaniço 2021 | Pediatric and adult patients with FVIII inhibitors | - | 9 | - | - | - | - | - | - | 0.0 | - | - |
| Cohen 2021 | Pediatric patients (≤18 years) with and without FVIII inhibitors | Q2W | 28 | 14.3 | - | - | 10.7 | 3.6 | 0.0 | - | - | - |
| Giuffrida 2021 | Patients with severe hemophilia A with and without FVIII inhibitors | QW, Q2W | 9 | 0.0 | 0.0 | 0.0 | 0.0 | 0.0 | 0.0 | 0.0 | - | - |
| Hassan, Jonathan and Jayashree 2021 | Pediatric patients (1–16.7 years) with severe hemophilia A with and without FVIII inhibitors | QW, Q2W, Q4W | 52 | - | - | - | 1.9 | 0.0 | 0.0 | 0.0 | 1.9 | 1.9 |
| Hassan and Motwani 2021 | Pediatric patients with and without FVIII inhibitors | - | 49 | - | 0.0 | - | - | 0.0 | 0.0 | 0.0 | 0.0 | 0.0 |
| Hassan and Motwani 2021 | Pediatric patients (0.72–13.2 years) with severe hemophilia with and without inhibitors | Q2W | 13 | - | - | - | - | - | - | 0.0 | - | - |
| Kenet 2021 | Pediatric patients with hemophilia A of all severities with and without FVIII inhibitors | - | 141 | 2.13 | - | - | 0.7 | 0.0 | 0.0 | 0.7§ | 0.7 | - |
| Lewandowska 2021 | Patients with and without FVIII inhibitors | - | 22 | - | - | - | - | 0.0 | 0.0 | 0.0 | - | - |
| Misgav 2021 | Adult (>50 years) patients with and without cardiovascular risk factors | - | 17 | - | 0.93 | - | - | 0.0 | 0.0 | 0.0 | - | - |
| Poon 2021 | Pediatric and adult patients with and without FVIII inhibitors | QW, Q2W, Other | 73 | - | - | - | - | 0.0 | 0.0 | - | - | - |
| Riera 2021 | Adult patients (20–48 years) with and without inhibitors | - | 5 | 20.0 | 20.0 | - | - | 0.0 | 0.0 | 0.0 | - | - |
| Schiavulli 2021 | Pediatric patients with severe hemophilia A with and without FVIII inhibitors | - | 18 | - | 0.0 | - | - | - | - | - | - | - |
| Swan 2021 | Pediatric patients (4–11 years) with severe hemophilia A with and without FVIII inhibitors | QW, Q2W | 10 | - | - | - | - | - | - | 0.0 | - | - |
| Warren 2021 | Pediatric and adult patients (0.55–79.8 years) with hemophilia A of all severities with previous or current FVIII inhibitors | QW, Q2W, Q4W, Other | 68 | 70.6 | - | - | - | 0.0 | 0.0 | 0.0 | - | - |
| Windyga 2021 | Adult patients (23–70 years) with FVIII inhibitors | - | 20 | - | - | - | - | 0.0 | 0.0 | 0.0 | - | - |
| Barg 2020 | Pediatric patients with severe hemophilia A with and without FVIII inhibitors | QW | 40 | - | - | - | 10.0 | 0.0 | 0.0 | 0.0 | - | - |
| Bush 2020 | Infants (1–23 months), PUPs and MTPs with severe hemophilia A | QW, Q4W | 6 | 16.7 | - | - | - | 0.0 | 0.0 | 0.0 | 0.0 | 0.0 |
| Catarino 2020 | Pediatric (4 months–12 years) and adult (27–55 years) patients with FVIII inhibitors | - | 17 | - | - | - | - | 0.0 | 0.0 | 0.0 | - | - |
| Ebbert 2020 | Pediatric and adult patients with hemophilia A of all severities with and without FVIII inhibitors | - | 42 | 11.9 | - | - | 7.1 | 0.0 | 2.4 | 0.0 | - | - |
| Garcia 2020 | Pediatric patients with severe hemophilia A with and without FVIII inhibitors | QW, Q2W | 30 | 0.0 | 0.0 | 0.0 | 0.0 | 0.0 | 0.0 | 0.0 | - | - |
| Lewandowska 2020 | Pediatric and adult (1–66 years) patients with moderate or severe hemophilia A with and without FVIII inhibitors | - | 122 | - | - | - | - | 0.0 | 0.0 | 0.0 | - | - |
| Linari 2020 | Patients with FVIII inhibitors | - | 7 | - | - | - | - | 0.0 | 0.0 | 0.0 | - | - |
| Ling 2020 | Patients with severe hemophilia A with and without FVIII inhibitors | QW, Q2W | 118 | - | - | - | - | - | - | - | 0.0 | 0.0 |
| McCary 2020 | Pediatric patients (<12 years) with and without FVIII inhibitors | QW, Q2W, Q4W | 93 | - | - | - | - | 0.0 | 0.0 | 0.0 | - | - |
| Mori 2020 | Infants (0.0–4.5 years), PUPs and PTPs with and without FVIII inhibitors | - | 17 | - | - | - | - | 0.0 | 0.0 | 0.0 | 0.0 | 0.0 |
| OngTang 2020 | Patients with severe hemophilia A with FVIII inhibitors | - | 8 | - | - | - | - | 0.0 | 0.0 | 0.0 | - | - |
| Shang 2020 | Patients with hemophilia A | - | 148 | 1.4 | - | - | - | 1.48\|\| | 0.0 | 0.0 | - | - |
| Vagrecha 2020 | Pediatric and adult patients with and without FVIII inhibitors | - | 38 | - | - | - | - | 0.0 | 0.0 | 0.0 | - | - |
| Wall 2020 | Patients with and without FVIII inhibitors | - | 172 | - | - | - | 4.6 | 1.74 | 0.0 | 1.2¶ | - | - |
| Barg 2019 | Infants with severe hemophilia A and FVIII inhibitors | - | 11 | - | - | - | - | 0.0 | 0.0 | 0.0 | - | - |

While every effort was made to ensure that the same patient was not captured more than once in these data, there is a potential risk that some individuals were included in more than one publication. *One patient died due to hemorrhagic shock secondary to a presumed gastrointestinal bleed (unrelated). ^†^One patient experienced a clot at the tip of the CVL (CVL-related thrombosis). ^‡^One patient experienced CVAD-related thrombosis. ^§^Unrelated problems. ^||^One patient aged >65 years receiving emicizumab and aPCC died due to a myocardial infarction. ^¶^One patient died due to multi-organ failure complicating a viral infection and one died due to intra-abdominal bleeding (delayed presentation to hospital), which was likely unrelated. Abbreviations: AE, adverse event; a PCC, activated prothrombin complex concentrate; CVAD, central venous access device; CVL, central venous line; FVIII, factor VIII; LISR, local injection-site reaction; MTP, minimally treated patient; PTP, previously treated patient; PUP, previously untreated patient; SAE, serious adverse event; TE, thrombotic event; TMA, thrombotic microangiopathy; TRAE, treatment-related adverse event.

**TABLE S8.** Safety case reports.

| **Author** | **Year** | **Patient age** | **FVIII inhibitors** | **Safety event** | **Related to emicizumab** | **Outcome** | **Treatment** |
| --- | --- | --- | --- | --- | --- | --- | --- |
| He et al. | 2022 | 13 | Yes | Traumatic hyphema | No | Resolved | Anterior vitrectomy and rFVIIa |
| Kawahara et al. | 2022 | 14 | Yes | Hemophilic pseudotumor | No | Resolved | Endoscopic resection |
| Capdevila et al. | 2021 | 25 | Yes | Inhibitor recurrence post-ITI | No | NR | NR |
| Doshi et al. | 2021 | 10 | Yes | Inhibitor recurrence post-ITI | No | Not resolved | Due to family preference  and only partial response to prior ITI, a repeat attempt at ITI was not initiated |
| George et al. | 2019 | 9 | No | Central line-associated VTE | No | Resolved | CVAD was removed and patient received two doses of rFVIII. Started enoxaparin while continuing emicizumab |
| Nagao et al. | 2019 | 50 | No | Right putamen hemorrhage | No | Resolved | rFVIIFc and tranexamic acid |
| Teo et al. | 2021 | Infant | Yes | Intracranial hemorrhage | No | Resolved | Initial daily FVIII, followed by burrhole drainage by neurosurgery |
| Barish & Hajdenberg | 2021 | 44 | No | Rectal bleeding | No | Resolved | rhFVIII and tranexamic acid |
| Gundabolu et al. | 2020 | 40 | Yes | STEMI and PE | Yes | Resolved | Myocardial infarction managed medically; DAPT initiated with metoprolol and atorvastatin. PE treated with supplemental oxygen and pain control |
| Chehade et al. | 2020 | 7 | Yes | Full-house lupus nephritis | Yes | Resolved | Spontaneous, full recovery 4 to 8 days after clinical presentation; initial hematuria and proteinuria also resolved spontaneously without further emicizumab but hematuria re-occurred on reintroduction of emicizumab |
| Wilson et al. | 2020 | 19 | Yes | Rhabdomyolysis | Yes | Resolved | Intravenous fluids and oral opioids, followed by "as needed" medication, including two doses of oral opioids |
| Riera et al. | 2021 | NR | No | Hematuria and a severe injection-site reaction | Yes | Not resolved | Emicizumab was stopped due to lack of effectiveness |
| Clark et al. | 2022 | 62 | NR | Intraparenchymal hemorrhage | Yes | Resolved | Human antihemophilic factor |
| Valke et al. | 2020 | 59 | Yes | Gastrointestinal bleeding | No | Resolved | rFVIII and tranexamic acid;  crystalloid fluids, red blood cell concentrates and pantoprazole;  coagulation with a gold probe and adrenaline |

Abbreviations: a, activated; CVAD, central venous access device; DAPT, dual-antiplatelet therapy; Fc, fusion protein; FVIII, factor VIII; ITI, immune tolerance induction; NR, not reported; PE, pulmonary embolism; r, recombinant; rh, recombinant human; STEMI, ST-segment elevation myocardial infarction; VTE, venous thromboembolism.

**TABLE S9.** Anti-drug antibody case reports.

| **Author** | **Year** | **Patient age** | **FVIII inhibitors** | **Type of ADA** | **Outcome** | **Treatment** |
| --- | --- | --- | --- | --- | --- | --- |
| Kaneda et al. | 2021 | Adult | Yes | Neutralizing | Not resolved | Emicizumab discontinuation due to a drop in emicizumab plasma levels to amounts insufficient to prevent bleeding |
| Peyvandi et al. | 2021 | 33 | No | Neutralizing | Resolved | ADA reduced on 7th dose of emicizumab and disappeared with 8th dose, with gradual recovery of emicizumab concentration up to 41 µg/mL in plasma |
| Harkins Druzgal et al. | 2020 | 6 | Yes | Neutralizing | Not resolved | Emicizumab discontinued and daily rFVIIa prophylaxis resumed |
| Ciavarella et al. | 2021 | 74 | Yes | Neutralizing | Not resolved | Emicizumab continued and no bleeding events observed |
| Harroche et al. | 2021 | 2 | Yes | ADA which induced increased emicizumab clearance | Not resolved | Emicizumab stopped after 3 months |

Abbreviations: ADA, anti-drug antibody; FVIII, factor VIII; rFVIIa, activated recombinant factor VII.
